# In vivo imaging of inflammatory response in cancer research

**DOI:** 10.1186/s41232-023-00261-x

**Published:** 2023-02-07

**Authors:** Yoshinobu Konishi, Kenta Terai

**Affiliations:** 1 Department of Medical Oncology, Dana-Farber Cancer Institute, Boston, MA USA; 2 Department of Pathology and Biology of Diseases, Graduate School of Medicine, Kyoto University, Kyoto, Japan

**Keywords:** Intravital microscopy, Two-photon excitation microscope, Tumor microenvironment, Förster resonance energy transfer, Optogenetics

## Abstract

Inflammation can contribute to the development and progression of cancer. The inflammatory responses in the tumor microenvironment are shaped by complex sequences of dynamic intercellular cross-talks among diverse types of cells, and recapitulation of these dynamic events in vitro has yet to be achieved. Today, intravital microscopy with two-photon excitation microscopes (2P-IVM) is the mainstay technique for observing intercellular cross-talks in situ, unraveling cellular and molecular mechanisms in the context of their spatiotemporal dynamics. In this review, we summarize the current state of 2P-IVM with fluorescent indicators of signal transduction to reveal the cross-talks between cancer cells and surrounding cells including both immune and non-immune cells. We also discuss the potential application of red-shifted indicators along with optogenetic tools to 2P-IVM. In an era of single-cell transcriptomics and data-driven research, 2P-IVM will remain a key advantage in delivering the missing spatiotemporal context in the field of cancer research.

## Background

Inflammation is part of the complex biological response triggered by the immune response to infection, injury, and other environmental challenges. Inflammation functions not only as a host defense process, but also as a tissue repair, regeneration, and remodeling process, modulating tissue homeostasis [1]. During the last couple of decades, it is increasingly appreciated that inflammation is an important component of tumorigenesis ranging from cancer development, progression, and therapy resistance [2]. Large epidemiological clinical studies clarifying the effects of non-steroidal anti-inflammatory drugs (NSAIDs) [3, 4] or interleukin 1β inhibitors [5] on reducing incidence and mortality in many cancer types have provided excellent evidence of the supportive role of inflammation in tumorigenesis. Defining the molecular and cellular cross-talks underlying tumor-promoting inflammation will be essential for further development of anti-cancer therapies.

Inflammatory responses are shaped by dynamic sequential inter-cellular cross-talks between a multitude of functionally diverse types of cells of innate and adaptive immunity. The development of high-dimensional profiling technologies such as cytometry by time of flight (CyTOF) and single-cell RNA sequencing (scRNA-seq) has enabled to profile of a vast heterogeneity of immune cell states and functionality. However, how different cellular components engage in the sequential inflammatory responses, and how these events take place in a spatially organized manner within the tumor microenvironment remain unsolved questions. Despite the recent advancement of organoid culture systems for modeling the tumor microenvironment [6, 7], recapitulation of inflammatory responses in vitro has yet to be achieved. A comprehensive understanding of this continuously dynamic and complex process requires in situ investigation in living organisms.

### Intravital microscopy with two-photon excitation microscopy (2P-IVM)

**Fig. 1:**
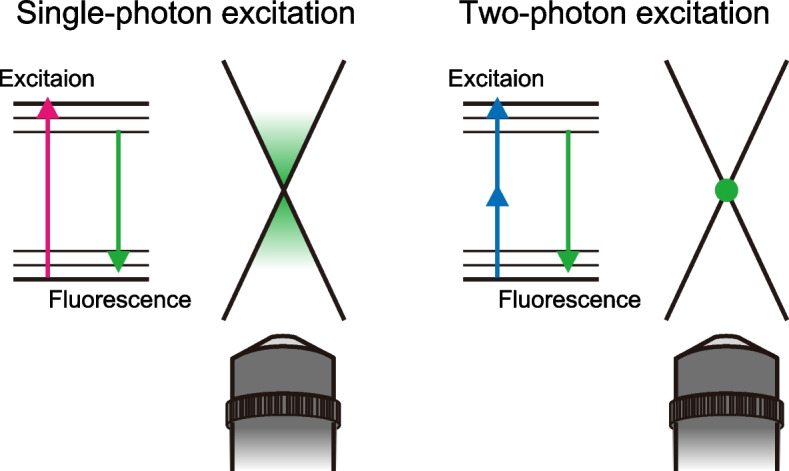
Properties of two-photon excitation microscopy (2PEM). In conventional single-photon fluorescence microscopy, fluorescence molecules along the light path are excited. In 2PEM, two photons must reach the single fluorophore almost simultaneously, which occurs only at the focal plane, markedly reducing the background fluorescence

Intravital microscopy (IVM) is a unique imaging method that allows observing biological processes in living organisms. The invention of the microscope leads the early pioneers to observe living cells in the seventeenth century [8]. Despite this long history, it was only after the widespread application of a two-photon excitation microscope (2PEM) that the number of studies using IVM started to increase [9, 10]. Two-photon excitation arises from the simultaneous absorption of two photons in a single event, which depends on the square of the light intensity. Due to this property, the probability of two-photon absorption at the center of focus is substantially greater than outside of the focus, allowing little excitation of fluorophores along the optical path in contrast to the conventional single-photon absorption (Fig. 1) [11]. This localization of two-photon excitation to solely the focal plane provides several advantages over conventional microscopy, including the maximum recovery of photons from the molecules of interest on focal planes, the reduction of photodamage throughout the sample, and the restricted out-of-focus absorption allowing deep tissue penetration [12]. With these important principles, 2PEM now allows us to observe brain tissue to more than 1-mm depth, in contrast to conventional microscopy, which can visualize only structures close to the surface of tissues [13].

Initially, after being introduced into the field of immunology, 2P-IVM provided significant insights into the dynamic cellular interplay between different fluorescently labeled cell populations [14–16]. Using pre-clinical tumor-bearing mouse models, 2P-IVM has revealed the inter-cellular cross-talks within the tumor microenvironment in numerous anatomical sites, including the skin [17, 18], lungs [19, 20], digestive tract [21], lymph nodes [22], bone marrow [23], and brain [24–26]. Also, the application of autofluorescence imaging along with fluorescent labeling that measures functional parameters like NAD(P)H and FAD for monitoring metabolic activity has provided an additional layer of information on immune cells [27].

Then, along with the development and application of fluorescent indicators, live signal transduction events now can be observed as cells actively engage in interactions or receive soluble stimuli [28]. Below, we will consider some applications of 2P-IVM with fluorescent indicators of signal transduction in the field of cancer research in detail.

### Visualization of PGE_2_ secretion from tumor cells

Prostaglandin E_2_ (PGE_2_) is the most widely produced prostanoid in the body, which can modulate various steps of inflammation in both pro-inflammatory and anti-inflammatory manners [29]. PGE_2_ is known to promote tumor development by several mechanisms that promote cell growth, invasion, migration, angiogenesis, and immune evasion [30–32]. Currently, it is believed that the concentration of PGE_2_ within tumor tissues is regulated mostly by transcriptional regulation of *Ptgs2* encoding cyclooxygenase-2 (COX-2) which is the rate-limiting enzyme in the PGE_2_ synthesis pathway [33]. However, the question remains of how the PGE_2_ secretion from tumor cells is regulated in the tumor microenvironment in the context of cellular cross-talks.

**Fig. 2:**
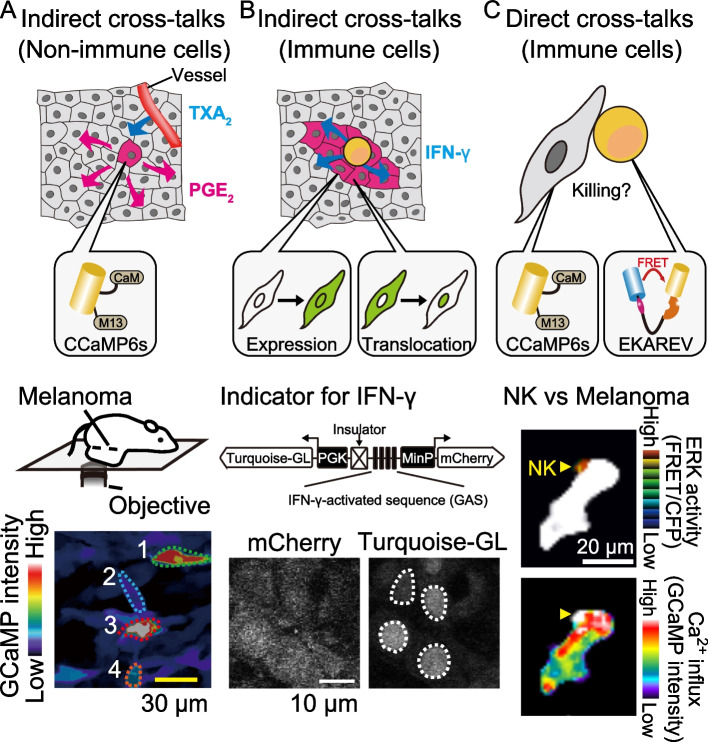
2P-IVM with fluorescent indicators to reveal the cross-talks between tumor cells and surrounding cells in the tumor microenvironment including both immune and non-immune cells. Indirect cross-talks between **A** tumor cells and non-immune cells, **B** tumor cells and immune cells, and **C** direct cross-talks between tumor cells and immune cells can be visualized by using 2P-IVM with fluorescent indicators (gray cells: tumor cells; magenta cells: tumor cells receiving signals via signaling molecules, TXA_2_ or IFN-γ; yellow cells: T cells or NK cells). Melanoma cells expressing GCaMP6s were imaged using 2P-IVM (**A**, bottom). The image represents the maximum intensity projection for 10 min. Images were adapted courtesy of Konishi et al. [36]. Schematic representation of the fluorescent indicator for IFN-γ (**B**, bottom). Melanoma cells expressing this fluorescent indicator were imaged using 2P-IVM. Images represent the mCherry and turquoise-GL-NLS of parental cells. Images were adapted by courtesy of Tanaka et al. [38]. Melanoma cells expressing GCaMP6s were imaged in the lung using 2P-IVM (**C**, bottom). Top, FRET/CFP ratio of an NK cell is shown. Melanoma cell is shown in white. GCaMP6s intensity is displayed in pseudo-color (bottom). The NK cell is shown in white. The images were adapted by courtesy of Ichise et al. [39]

To monitor PGE_2_ secretion using 2P-IVM, we focused on Ca^2+^ signal transduction in tumor cells. The Ca^2+^-induced activation of phospholipase A1 (PLA2), particularly cytosolic phospholipase A2 (cPLA2a), is recognized as the rate-limiting step in PGE_2_ secretion, which liberates arachidonic acid from cell membrane phospholipids [34, 35]. Therefore, we hypothesized that the Ca^2+^ transients in tumor cells can work as a surrogate marker for PGE_2_ secretion [36]. The Ca^2+^ transients in mouse melanoma cells in the tumor microenvironment were visualized by using a genetically encoded calcium indicator, GCaMP6s (Fig. 2A) [37]. Of note, the Ca^2+^ transients observed in tumor cells were significantly suppressed by CRISPR/Cas9-mediated gene knockout of Gnaq (*Gnaq*^*−/−*^), which encodes guanine nucleotide-binding protein G(q) subunit alpha. Importantly, this suppression in Ca^2+^ transients resulted in a marked reduction of the PGE_2_ concentration in the tumor microenvironment, supporting the notion that (i) Ca^2+^ transients reflect PGE_2_ secretion and (ii) the GqPCR signaling pathway is required for PGE_2_ secretion from tumor cells in this melanoma model. The downstream analysis revealed that the major GqPCR ligand in this model was thromboxane A2 (TXA_2_) released from endothelial cells. As shown here, 2P-IVM with the fluorescent indicator of Ca^2+^ signal transduction enabled us to clarify the indirect intercellular cross-talks between tumor cells and endothelial cells in enhancing PGE_2_ secretion from tumor cells and thereby promoting tumor immune evasion within the tumor microenvironment.

### Visualization of IFNγ-induced signaling in tumor cells

Using the same mouse melanoma model with 2PM-IVM, Tanaka et al. successfully visualized the tumor cells receiving interferon-gamma (IFN-γ) stimuli in the tumor microenvironment [38]. IFN–γ is a cytokine secreted by immune cells, especially NK cells and T cells, and enhances anti-tumor immune response [40, 41]. Tanaka et al. utilized the dual promoter system with interferon γ-activated sequence (GAS) [42] and developed a fluorescent indicator for IFN-γ-mediated signal transduction in tumor cells (Fig. 2B). This indicator enabled the visualization of tumor cells receiving IFN-γ stimuli as the expression of fluorescent protein at a single-cell resolution. As anticipated, the GAS activity in the *Gnaq*^*−/−*^ tumor was significantly higher than that in the parental tumors, supporting the activated anti-tumor immune response with an abundance of IFN-γ. Similarly, Hoekstra et al. analyzed the spatial spreading of T cell-derived IFN-γ in vivo by using an IFN-γ-sensing fluorescent indicator that induces the expression of fluorescent protein after IFN-γ receptor triggering [42]. In addition to these two examples, the spatiotemporal spread of IFN-γ within the tumor microenvironment was also visualized by a fluorescent indicator where the translocation of a fluorescent protein from the cytoplasm to the nucleus represents the receiving of IFN-γ stimuli [43]. These prominent examples support the utility of 2P-IVM in recording indirect cellular cross-talks via soluble stimuli such as IFN-γ.

### Visualization of tumor killing by NK cells

To develop optimal immunotherapies, it is important to understand how cytotoxic lymphocytes such as NK cells react to tumor cell challenges and how tumor cells in turn respond. Using 2P-IVM with a metastatic melanoma mouse model, Ichise et al. visualized the direct intercellular cross-talks between NK cells and metastatic tumor cells in the lung (Fig. 2C) [39]. To track NK cell activation, extracellular signal-regulated kinase (ERK) activity was monitored by using a Förester resonance energy transfer (FRET)-based biosensor (EKAREV) [44, 45]. In the meantime, Ca^2+^ influx in tumor cells was monitored by using GCaMP6s [46] as a surrogate marker for apoptosis. Using this system, they revealed that ERK activation in NK cells contributes to tumor-cell killing, granting further study for underlying mechanisms controlling ERK activity dynamics in NK cells to develop optimal immunotherapies using NK cells. Interestingly, they also reported a marked decrease in the proportion of ERK activation and Ca^2+^ influx 24 h after tumor injection, suggesting exhaustion of NK cells.

These examples indicate the versatile utility of 2P-IVM with a fluorescent indicator of signal transduction in analyzing inflammatory responses in the tumor microenvironment, encouraging the application of 2P-IVM in the field of cancer research.

### Future perspective

Recent advances in optogenetics have opened new routes to cancer research with 2P-IVM. The term “optogenetics” was first coined by Deisseroth et al. in 2006 [47]. Optogenetics is a biological technique that employs natural and engineered photoreceptors to be genetically introduced into the cells of interest, allowing these target cells to be photosensitive and addressable by illumination [48–50]. On the level of individual cells, this allows the precise control of cell signaling pathways in time and space. Starting with Channelrhodopsin (ChR) to excite neurons as a prototypical optogenetic tool [51, 52], several optogenetic tools are now available to control the activity of neurons or other cell types with light by regulating protein heterodimerization [53–55], homo-dimerization [56, 57], gene expression [58–60], protein degradation [61], nuclear-cytosolic protein translocation [62–65], and liquid–liquid protein phase separation [66–68]. Optogenetic manipulation can be achieved by two-photon excitation and is compatible with 2P-IVM. For example, the optogenetic control of protein dimerization to induce signal transduction and gene expression has been achieved by two-photon illumination [69–71]. Indeed, Kinjo et al. achieved the simultaneous photoactivation and recording of ERK signaling pathway activity in the mouse epidermal cells at a single-cell resolution during 2P-IVM by introducing both a fluorescent indicator and an optogenetic tool. The recent development of red, near-infrared, and far-red shifted fluorescent indicators including calcium [72, 73], voltage [74, 75], and kinase activity [76] has strengthened the potential for accomplishing 2P-IVM in combination with optogenetic control. The application of red-shifted indicators along with optogenetic tools to 2P-IVM will advance the utility of this versatile tool in the field of cancer research.

## Conclusions

In the field of cancer research, major challenges remain in elucidating the dynamic sequential intercellular cross-talks within the tumor microenvironment. The 2P-IVM with fluorescent indicators of signal transduction enables us to investigate this continuously dynamic and complex process in living organisms in situ and real-time. The application of red-shifted indicators along with optogenetic tools will further facilitate the potential of 2P-IVM, revealing unexpected cellular and molecular mechanisms operating in the tumor microenvironment.

## Data Availability

Not applicable.

## Abbreviations

2P-IVM: Intravital microscopy with two-photon excitation microscopes
NSAIDs: Non-steroidal anti-inflammatory drugs
CyTOF: Cytometry by the time of flight
scRNA-seq: Single-cell RNA sequencing
2PEM: Two-photon excitation microscope
PGE_2_: Prostaglandin E_2_
PLA2: Phospholipase A1
cPLA2a: Cytosolic phospholipase A2
TXA_2_: Thromboxane A2
IFN–γ: Interferon-gamma
ERK: Extracellular signal-regulated kinase
FRET: Förester resonance energy transfer
ChR: Channelrhodopsin
